# Frequency-Offset-Estimation-Assisted Transformer Neural Equalization for a 4.6 km Optical-Heterodyne RoF–Wireless OFDM Link

**DOI:** 10.3390/s26175615

**Published:** 2026-09-03

**Authors:** Zhihang Ou, Wen Zhou, Ye Zhou, Jiali Chen, Xin Lu, Hansong Ma, Sicong Xu, Jie Zhang, Hanyu Zhang, Yubin Zhang, Jianjun Yu

**Affiliations:** State Key Laboratory of ASIC and System, Key Laboratory for Information Science of Electromagnetic Waves (MoE), School of Information Science and Technology, Fudan University, Shanghai 200433, China; 26113090149@m.fudan.edu.cn (Z.O.); zwen@fudan.edu.cn (W.Z.); 24110720111@m.fudan.edu.cn (Y.Z.); 25113090124@m.fudan.edu.cn (J.C.); 25113090101@m.fudan.edu.cn (X.L.); 23110720118@m.fudan.edu.cn (H.M.); 24110720169@m.fudan.edu.cn (S.X.); 25113090116@m.fudan.edu.cn (J.Z.); 24210720317@m.fudan.edu.cn (H.Z.); 24210720074@m.fudan.edu.cn (Y.Z.)

**Keywords:** OFDM, radio-over-fiber (RoF), optical heterodyne, frequency offset estimation (FOE), Transformer, neural-network equalizer, fiber-wireless transmission

## Abstract

To address the issues of subcarrier orthogonality loss and inter-carrier interference (ICI) caused by carrier frequency offset (CFO), this paper proposes and experimentally validates a frequency offset estimation (FOE)-assisted dual-domain Transformer equalizer within an advanced, high-capacity optical-heterodyne radio-over-fiber (RoF)–wireless orthogonal frequency division multiplexing (OFDM) transmission system. To rigorously test the algorithm’s robustness under extreme physical conditions, the experimental platform integrates offline 16-GBaud signal generation, optical I/Q modulation, dual-optical-tone transport over a single-mode-fiber RoF feeder, remote photonic heterodyne frequency conversion based on a uni-traveling-carrier photodiode (UTC-PD), 4.6 km free-space wireless transmission, and 160-GSa/s ultra-high-speed real-time sampling. In this system, the receiver front-end employs an FOE module to pre-compensate for the dominant global CFO-induced phase rotation; subsequently, a low-complexity, compact local-window Transformer is utilized to perform adaptive residual compensation for local data-dependent impairments—such as residual waveform distortion and residual ICI—in both the time and frequency domains (before and after the Fast Fourier Transform, or FFT). This synergistic architecture, combining a physical model-driven approach with a self-attention mechanism, effectively mitigates the adverse impact of global frequency offset on neural network convergence. Experimental results demonstrate that, under conditions of strictly aligned multiply accumulate (MAC) operation complexity, the dual-domain architecture achieves significantly superior performance—in terms of bit error rate (BER), error vector magnitude (EVM), and constellation quality—compared to traditional linear DSP methods and baseline networks such as DNNs, CNNs, and LSTMs. Operating in 16 GBaud QPSK mode with an input optical power of 0 dBm, the system achieves a BER of $1.89\times 10^{-4}$, representing performance improvements of approximately 5.98-fold and 1.92-fold over the standalone Transformer and FOE-assisted DNN schemes, respectively.

## 1. Introduction

Photonic radio-over-fiber (RoF) and photonic-assisted wireless transmission have attracted increasing interest for high-capacity access, fronthaul, backhaul, and future beyond-5G/6G communication systems [1]. In an optical-heterodyne RoF architecture, an information-bearing optical carrier and a second optical tone can be transported over fiber to a remote photodetector, where their frequency spacing is converted directly into a millimeter-wave or terahertz carrier. This centralized optical generation and fiber distribution reduces the high-frequency electronic burden at the remote radio unit while retaining broad and tunable carrier-generation capability [2,3]. Orthogonal frequency-division multiplexing (OFDM) is a promising modulation format for such RoF–wireless links because it divides a frequency-selective broadband channel into multiple narrowband subcarriers and enables efficient frequency-domain equalization after a fast Fourier transform (FFT) [4,5].

However, OFDM is intrinsically sensitive to carrier frequency offset (CFO) [6,7]. In a practical optical-heterodyne RoF–wireless link, CFO may originate from relative optical-carrier drift, heterodyne instability at the photodetector, electrical local-oscillator mismatch, residual sampling or synchronization error, and environmental fluctuation during free-space propagation. A residual CFO introduces a sample-dependent phase rotation across the OFDM symbol. After FFT, this effect destroys the approximately diagonal subcarrier-domain channel model and causes inter-carrier interference (ICI). Therefore, frequency offset estimation (FOE) and compensation should be performed before reliable frequency-domain equalization and symbol decision [8,9].

Conventional receiver-side DSP for OFDM usually follows a physically ordered processing chain, including resampling, frame synchronization, FOE, cyclic-prefix removal, FFT, channel equalization, phase recovery, and symbol decision. These model-based operations are efficient and interpretable, but they are often insufficient for measured RoF–wireless links. In addition to CFO, the received signal may contain residual timing error, phase fluctuation, electro-optic modulator distortion, fiber-distribution effects, front-end bandwidth limitation, I/Q imbalance, amplifier nonlinearity, UTC-PD photodetection imperfection, mixer distortion, and free-space fading. These cascaded optical, electrical, and wireless impairments are coupled in time and frequency domains and are difficult to fully compensate using only conventional linear DSP [10].

Neural-network-based equalizers have therefore been investigated as data-driven tools for optical, RoF, and photonic-wireless communication systems. Fully connected deep neural networks (DNNs), convolutional neural networks (CNNs), and long short-term memory networks (LSTMs) can learn nonlinear inverse mappings from measured data. Nevertheless, their structures are not always well matched to FOE-assisted OFDM residual equalization. DNNs do not explicitly exploit neighboring sample or subcarrier relationships unless such context is manually provided. CNNs can model local patterns but rely on fixed convolution kernels, which limits their adaptability to data-dependent ICI and phase distortion. LSTMs can capture sequence memory, but their recurrent structure is less parallel and may be less attractive for high-throughput receiver implementation [11,12].

The Transformer architecture provides a more flexible alternative because its self-attention mechanism can adaptively assign weights to neighboring samples or subcarriers within a local processing window [13]. This property is particularly useful after FOE. Once the dominant global CFO-induced phase rotation has been compensated, the remaining impairments are mainly local, structured, and data-dependent, such as residual ICI, subcarrier-dependent phase error, amplitude distortion, and hardware-induced nonlinear residuals. In this regime, the Transformer does not need to learn the full deterministic CFO from scratch. Instead, it can focus its model capacity on residual time-frequency equalization [14]. This explains why FOE and Transformer are more complementary than FOE and conventional neural equalizers.

However, Transformer equalizers usually have higher computational complexity than DNN, CNN, or LSTM baselines because multi-head self-attention and feed-forward blocks involve multiple matrix operations [15,16]. To make the proposed method practical for receiver-side DSP, the Transformer should not be used as an oversized black-box neural receiver. Instead, compact local-window Transformers with small embedding dimensions, limited encoder depths, and complexity-matched configurations are adopted. In this way, the performance gain can be attributed to FOE-assisted self-attention-based residual learning rather than to a substantially larger number of multiply-accumulate operations [17,18].

Another important design issue is where the Transformer should be inserted in the OFDM DSP chain. A time-domain Transformer before cyclic-prefix removal and FFT can improve the quality of the received waveform by mitigating waveform-level residual distortion, timing-induced memory, phase fluctuation, and front-end bandwidth effects. A frequency-domain Transformer after FFT can directly refine the subcarrier symbols and suppress residual ICI, subcarrier-dependent phase error, and amplitude distortion [10]. Since the two domains correspond to different physical stages of OFDM reception, using only one of them may leave part of the residual impairment uncompensated. Therefore, this work adopts a dual-domain Transformer structure: the time-domain Transformer prepares a cleaner waveform for FFT, and the frequency-domain Transformer further improves the recovered subcarrier symbols [18].

In this paper, we propose and experimentally demonstrate an FOE-assisted dual-domain Transformer equalizer for receiver-side DSP in a 4.6 km optical-heterodyne RoF–wireless OFDM transmission system. In the RoF portion, an optically modulated OFDM carrier and an unmodulated optical tone are combined, transported over single-mode fiber (SMF), and heterodyned in a uni-traveling-carrier photodiode (UTC-PD) to generate the high-frequency wireless carrier near the transmitting antenna. The resulting OFDM signal is then delivered over a 4.6 km free-space wireless path. The proposed receiver follows the physical processing order of OFDM reception, where cyclic-prefix-correlation-based FOE first compensates the dominant global CFO, and time- and frequency-domain Transformers subsequently learn residual impairments before and after FFT, respectively. By combining model-driven CFO compensation with data-driven dual-domain residual equalization, the proposed scheme effectively improves waveform quality, suppresses residual ICI, and enhances symbol recovery. Experimental results in terms of BER, EVM, constellation, complexity, convergence, and hyperparameter analysis verify that the proposed method achieves the best overall performance compared with traditional DSP, DNN, CNN, LSTM, Transformer-only, and FOE-assisted DNN baselines under comparable computational complexity. The primary novelty of this work lies in this ordered combination: rather than treating the entire impairment compensation as a black-box neural network task, the physical FOE module explicitly handles the well-known deterministic CFO, empowering the dual-domain Transformer to dedicate its self-attention learning capacity strictly to complex, non-linear residual distortions.

## 2. Principle of FOE-Assisted Transformer Equalization

### 2.1. OFDM Signal Model with Carrier-Frequency Offset

For an OFDM symbol with useful IFFT size *N*, the transmitted baseband samples can be written as

(1) $$s[n]=\frac{1}{\sqrt{N}}\sum_{k=0}^{N-1}X[k]e^{j\frac{2\pi kn}{N}},n=0,1,\ldots ,N-1$$

where *X*[*k*] denotes the complex modulation symbol on the *k*-th subcarrier. After transmission through the cascaded optical-heterodyne RoF and wireless channel, the received sequence can be expressed as

(2) $$r[n]=h[n]*s[n]e^{j\frac{2\pi \varepsilon n}{N}}+w[n]$$

where *h*[*n*] is the equivalent channel impulse response, *w*[*n*] is additive noise, and epsilon is the CFO normalized to the subcarrier spacing. The exponential term creates a sample-index-dependent phase ramp. After FFT, the received subcarrier is no longer a purely diagonal channel output; instead, it includes leakage from neighboring subcarriers:

(3) $$Y[k]=H[k]X[k]+\sum_{\begin{array}{l} m=0\\ m\neq k \end{array}}^{N-1}I_{k,m}(\varepsilon )H[m]X[m]+W[k]$$

The ICI kernel $I_{k,m}(\varepsilon )$ depends on the residual frequency offset. If a neural equalizer directly processes *r*[*n*] or *Y*[*k*] without CFO compensation, it must jointly learn a global deterministic phase ramp, channel response, residual ICI, nonlinear distortion, and noise. The method we proposed reduces this learning burden by moving the dominant CFO correction to an explicit FOE front end.

### 2.2. Cyclic-Prefix-Correlation-Based FOE

The cyclic prefix (CP) is a copy of the end of the useful OFDM symbol and is separated from the corresponding tail samples by N samples. The phase difference between these repeated samples is mainly determined by the normalized CFO. For a CP length *N_CP_*, the correlation metric is

(4) $$C=\sum_{n=0}^{N_{CP}-1}r[n+N]r^{*}[n]$$

The normalized frequency offset is estimated by

(5) $$\hat{\varepsilon }=\frac{1}{2\pi }\arg(\sum_{n=0}^{N_{CP}-1}r[n+N]r^{*}[n])$$

(6) $$\tilde{r}[n]=r[n]e^{-j\frac{2\pi \hat{\varepsilon }n}{N}}$$

When the residual offset $∆\varepsilon =\varepsilon -\hat{\varepsilon }$ is small, the compensated OFDM waveform is closer to the diagonal subcarrier model. This does not remove all impairments, but it changes the neural network learning problem from full CFO and ICI to residual impairment learning after physical normalization.

### 2.3. Transformer Residual Equalizer

**Figure 1:**
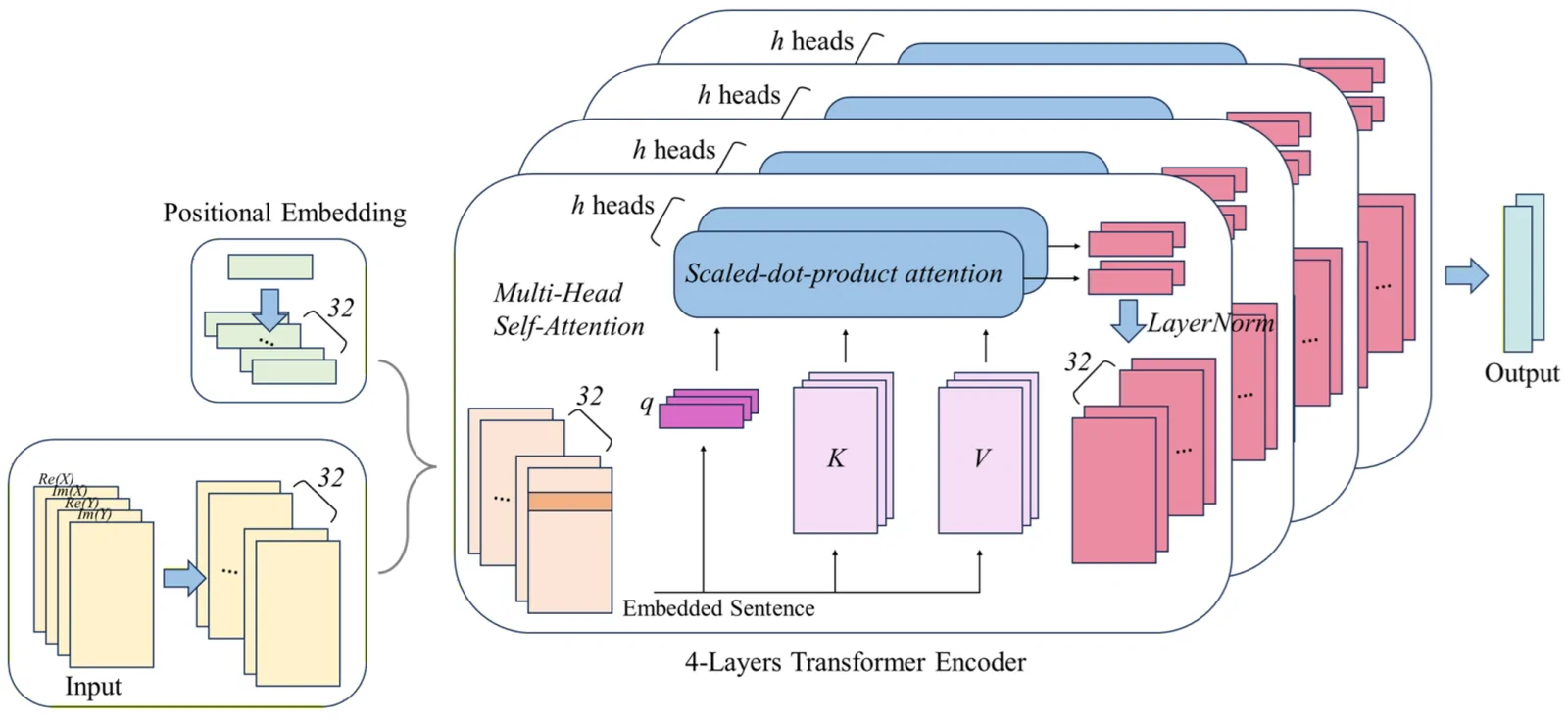
Transformer neural-network equalizer architecture used for receiver-side OFDM signal recovery.

The Transformer equalizer uses the architecture shown in Figure 1. The complex input is represented by in-phase and quadrature components. For each target time sample or subcarrier, a local window is extracted, linearly embedded into a *d_model_* dimensional representation, and combined with positional embedding so that the network can distinguish the order of samples or subcarriers.

For an embedded sequence *Z*, the scaled dot-product attention in each head is

(7) $$Attention(Q,K,V)=softmax(\frac{QK^{T}}{\sqrt{d_{k}}})V$$

where *Q*, *K*, and *V* are learned query, key, and value projections. Multi-head attention allows different heads to emphasize different impairment relationships, such as adjacent-subcarrier leakage, asymmetric filter response, residual phase fluctuation, or power-dependent nonlinear distortion. The Transformer output is formulated as a residual correction:

(8) $$\hat{x}=y+F_{\theta }(y)$$

where *y* is the FOE-compensated input window and $F_{\theta }(\cdot )$ is the learned residual mapping. Residual learning is especially suitable after FOE because the remaining correction is smaller, smoother, and more local in the time-frequency plane.

### 2.4. Cooperation Between FOE and Self-Attention

**Figure 2:**
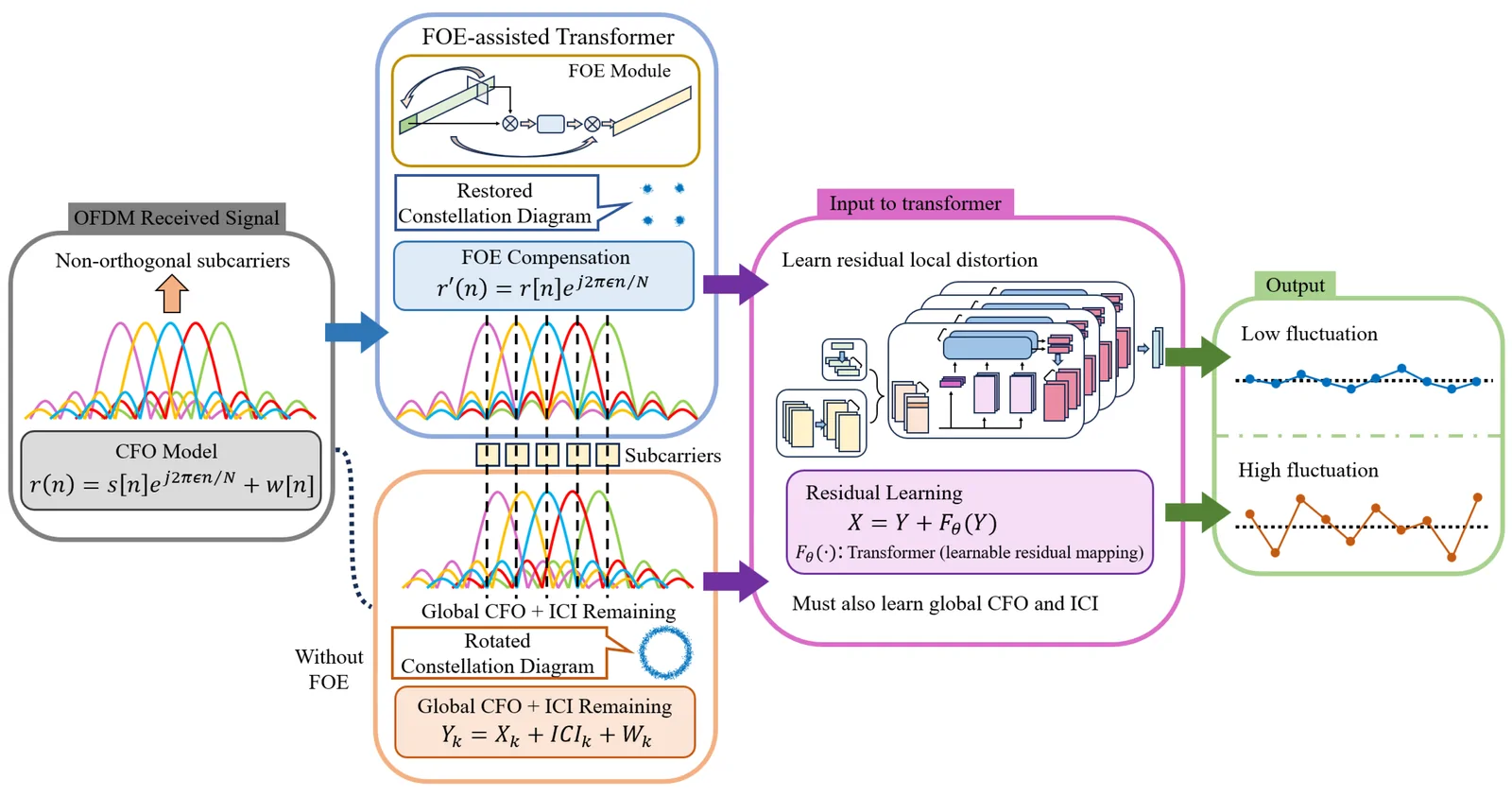
Cooperation between CP-correlation-based FOE and Transformer residual equalization for OFDM signal recovery.

Figure 2 illustrates the operating principle of the proposed FOE-assisted Transformer equalizer. In the received OFDM waveform, the dominant carrier frequency offset introduces a sample-dependent phase rotation, which appears as a global phase ramp across the OFDM symbol and destroys the orthogonality among subcarriers. If this impairment is directly fed into a neural equalizer, the network has to learn both the deterministic CFO-induced rotation and the residual channel distortions, resulting in a more difficult and less stable learning task. In the proposed receiver, the CP-correlation-based FOE module first estimates the dominant CFO from the repeated structure of the cyclic prefix and compensates the corresponding phase rotation in the time domain. After this model-driven CFO compensation, the input to the Transformer is no longer dominated by the global frequency-offset impairment. The remaining errors mainly consist of residual ICI, phase fluctuation, bandwidth limitation, device nonlinearity, and local time-frequency distortions. Therefore, the Transformer equalizer can focus on learning a residual correction within a neighboring sample or subcarrier window. Therefore, lower signal fluctuations can be obtained. This decomposition explains why FOE and Transformer equalization are complementary: FOE removes the dominant global CFO, while the self-attention mechanism learns the remaining structured residual impairments.

## 3. Experimental RoF-Wireless Setup and Receiver DSP

### 3.1. Optical-Heterodyne RoF-Wireless OFDM Transmission System

**Figure 3:**
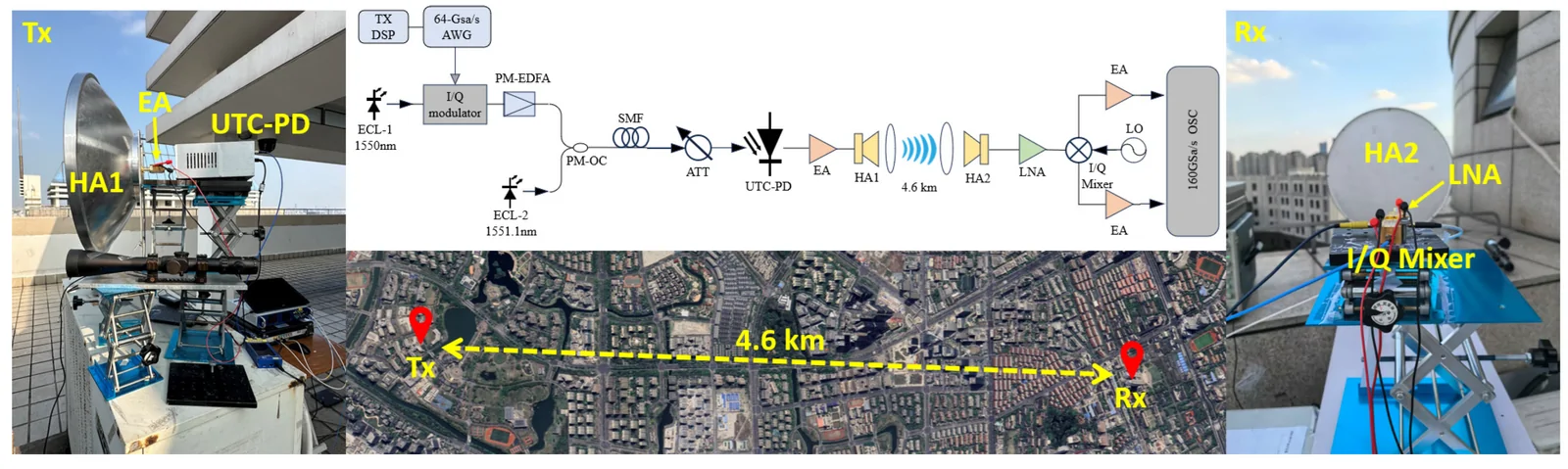
Experimental optical-heterodyne RoF–wireless OFDM transmission setup with an SMF feeder and a 4.6 km free-space link.

Figure 3 shows the experimental optical-heterodyne RoF–wireless OFDM transmission system, which comprises a central optical transmitter, an SMF RoF feeder with a remote UTC-PD photonic upconverter, a 4.6 km free-space wireless link, and an electrical receiver. At the central transmitter, the OFDM waveform is generated offline by symbol mapping, pilot insertion, subcarrier allocation, IFFT, cyclic-prefix insertion, and frame assembly. In the experiment, each OFDM frame contains 1024 subcarriers, among which 440 subcarriers are effectively loaded. Six OFDM symbols are arranged in one frame, and a cyclic prefix (CP) with a length of 64 samples is inserted to maintain the circular-convolution property after transmission. QPSK modulation is adopted, and comb-type pilots are inserted every eight subcarriers to support synchronization, channel monitoring, and performance evaluation. The generated complex I/Q samples are uploaded to a 64-GSa/s arbitrary waveform generator and applied to an optical I/Q modulator driven by ECL-1 at 1550 nm.

The RoF portion begins with the optical I/Q modulation of ECL-1. The resulting 16-GBaud OFDM-modulated optical carrier is amplified by a polarization-maintaining EDFA (PM-EDFA), combined in a polarization-maintaining optical coupler (PM-OC) with an unmodulated tone from ECL-2 at 1551.1 nm, and delivered through SMF to the remote UTC-PD; an optical attenuator controls the UTC-PD input power. Thus, both the information-bearing optical carrier and the optical local-oscillator tone are distributed in the optical domain. At the remote radio end, the UTC-PD performs optical-to-electrical conversion and photonic heterodyne upconversion simultaneously. The approximately 1.1 nm wavelength separation produces a tunable carrier of about 137 GHz in the D band, on which the 16-GBaud OFDM waveform is carried. This RoF architecture avoids the need for a 137-GHz electronic oscillator or cascaded frequency multipliers at the remote unit. After electrical amplification, the generated D-band OFDM signal is radiated by the transmitting antenna HA1 and propagated over a 4.6 km free-space wireless path.

**Figure 4:**
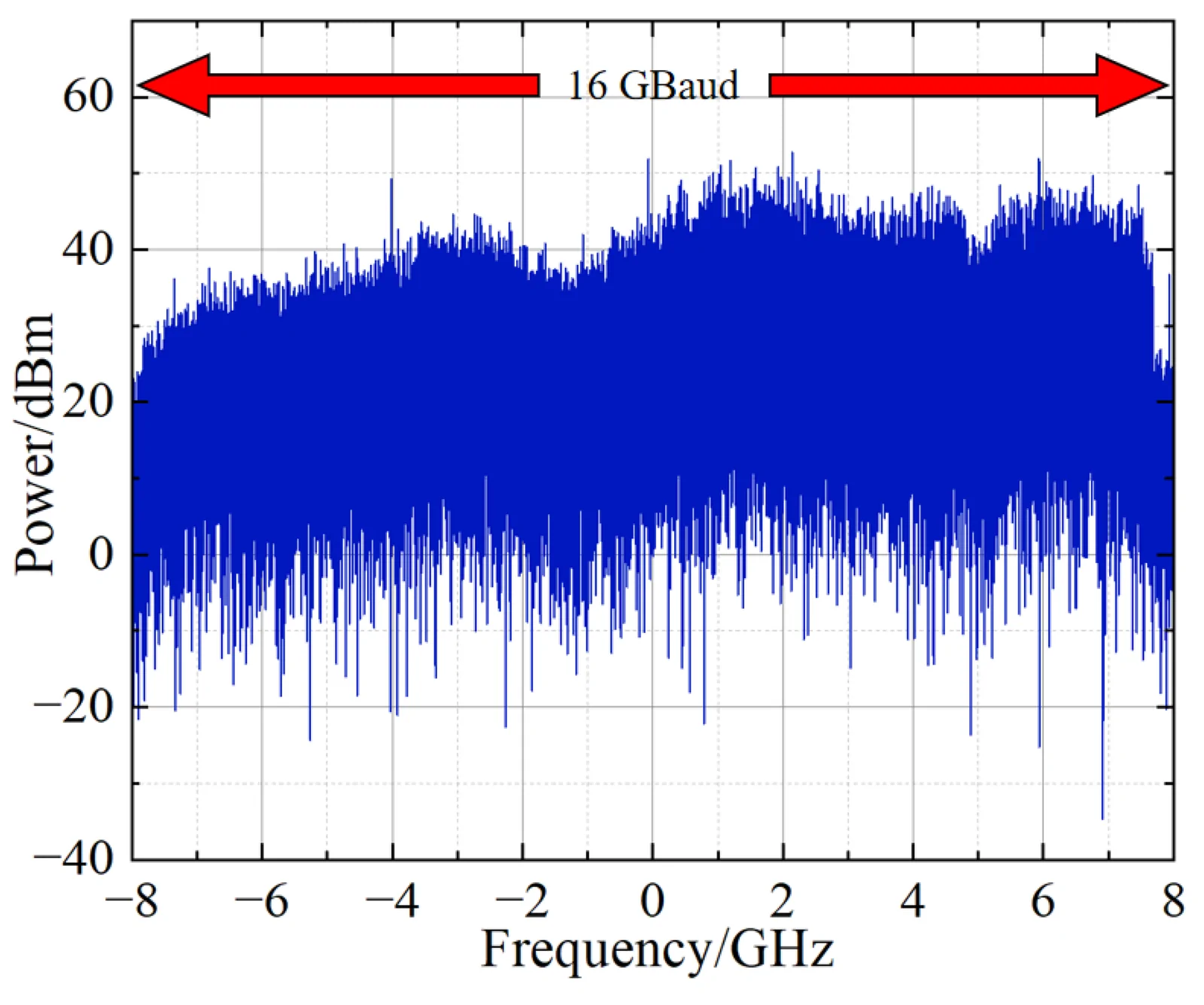
Spectrum of the received OFDM signal.

At the wireless receiver, antenna HA2 collects the D-band signal, which is amplified by a low-noise amplifier and down-converted by an I/Q mixer driven by an electrical local oscillator. The down-converted I/Q waveform is electrically amplified, captured by a 160-GSa/s real-time oscilloscope, and processed offline. As a reference for the signal quality prior to further receiver processing, Figure 4 shows the spectrum of the received OFDM signal.

### 3.2. Ordered FOE-Assisted Dual-Domain Receiver DSP Chain

**Figure 5:**
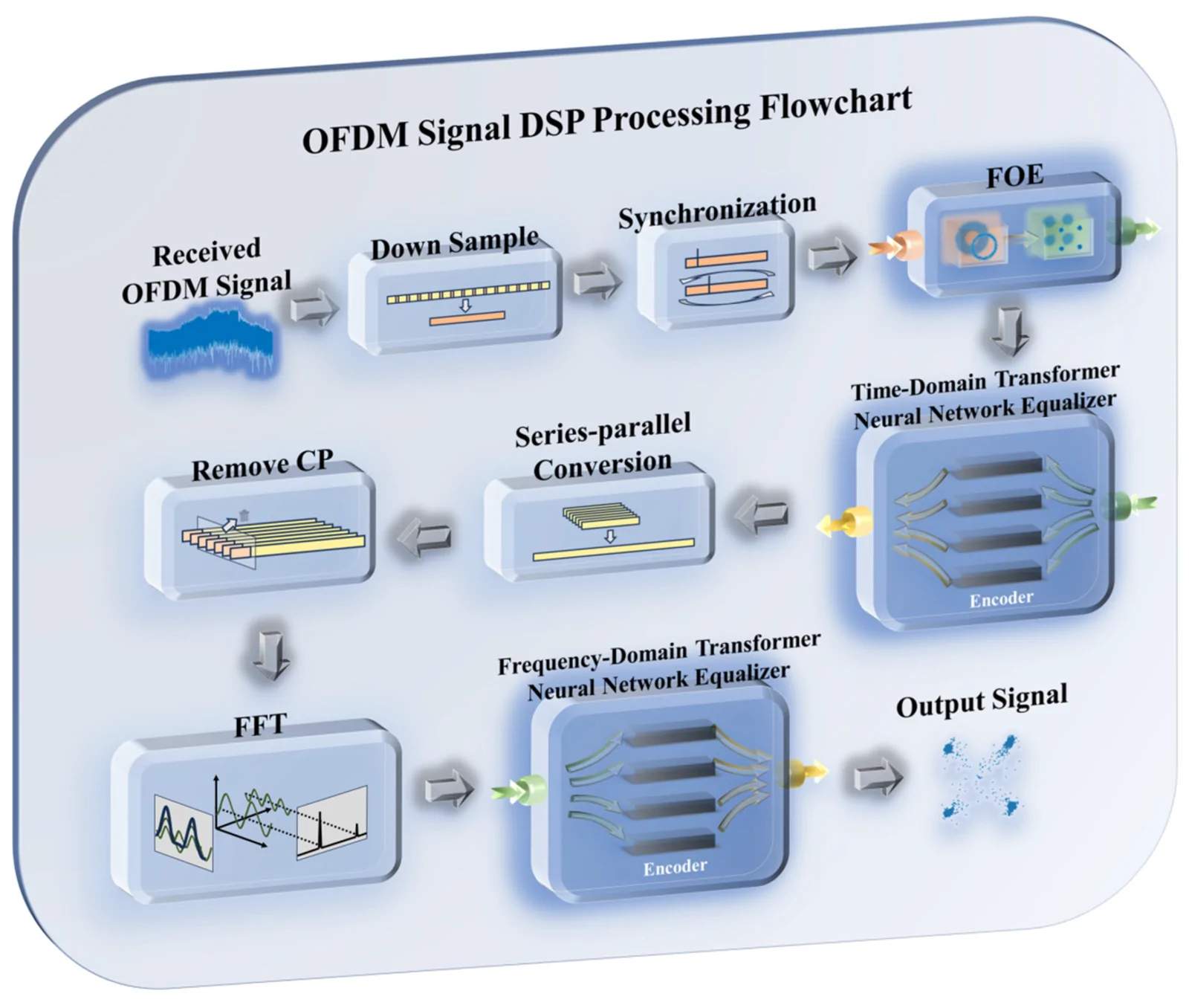
Receiver DSP flow. Including down-sampling, synchronization, FOE, time-domain Transformer equalization, serial-parallel conversion, CP removal, FFT, and frequency-domain Transformer equalization.

The receiver DSP chain is shown in Figure 5. The captured waveform is first down-sampled, frame-synchronized, and aligned to the OFDM symbol boundary. FOE is then performed in the time domain using the CP correlation in (4)–(6). After FOE compensation, the waveform is sent to the time-domain Transformer.

The output of the time-domain Transformer is then converted from serial to parallel format, the CP is removed, and an FFT is performed. The frequency-domain Transformer operates on neighboring subcarrier windows after FFT.

The main algorithmic innovation is the ordered cooperation among CP-correlation-based FOE, time-domain self-attention, and frequency-domain self-attention. FOE first removes the dominant global CFO component with a low-complexity physical estimator. The time-domain Transformer then learns residual waveform corrections, and the frequency-domain Transformer learns residual subcarrier corrections. Compared with a pure neural receiver, this design gives the neural networks a better-conditioned input. Compared with FOE-assisted DNN equalization, the Transformer can adaptively model local sample and subcarrier dependencies through attention weights rather than relying on fixed, fully connected mappings.

### 3.3. Neural-Network Equalizer Configurations and Complexity Control

Table 1 summarizes the neural equalizer configurations used for fair comparison. The proposed Transformer uses four encoder layers, two attention heads, and an embedding dimension of 32. The time-domain branch uses a 9-sample local window and a feed-forward dimension of 16, while the frequency-domain branch uses an 11-subcarrier local window and a feed-forward dimension of 32. These settings are selected to provide enough representation capability for residual impairment learning while keeping the receiver complexity within a practical range. For a fair evaluation, the DNN, CNN, and LSTM baselines are configured with similar multiply-accumulate (MAC) operation counts in both domains.

This complexity-controlled design is important for advanced optical-heterodyne RoF–wireless receivers. The receiver must process broadband OFDM waveforms affected by the cascaded RoF feeder, photonic upconverter, and long-range wireless channel, and it cannot rely on unlimited neural-network capacity. By limiting attention to local windows and using compact encoder dimensions, the proposed algorithm keeps the MAC counts comparable to conventional neural equalizers while still exploiting the adaptive dependency modeling capability of Transformer self-attention.

## 4. Experimental Results and Discussion

### 4.1. BER and Constellation Performance

**Figure 6:**
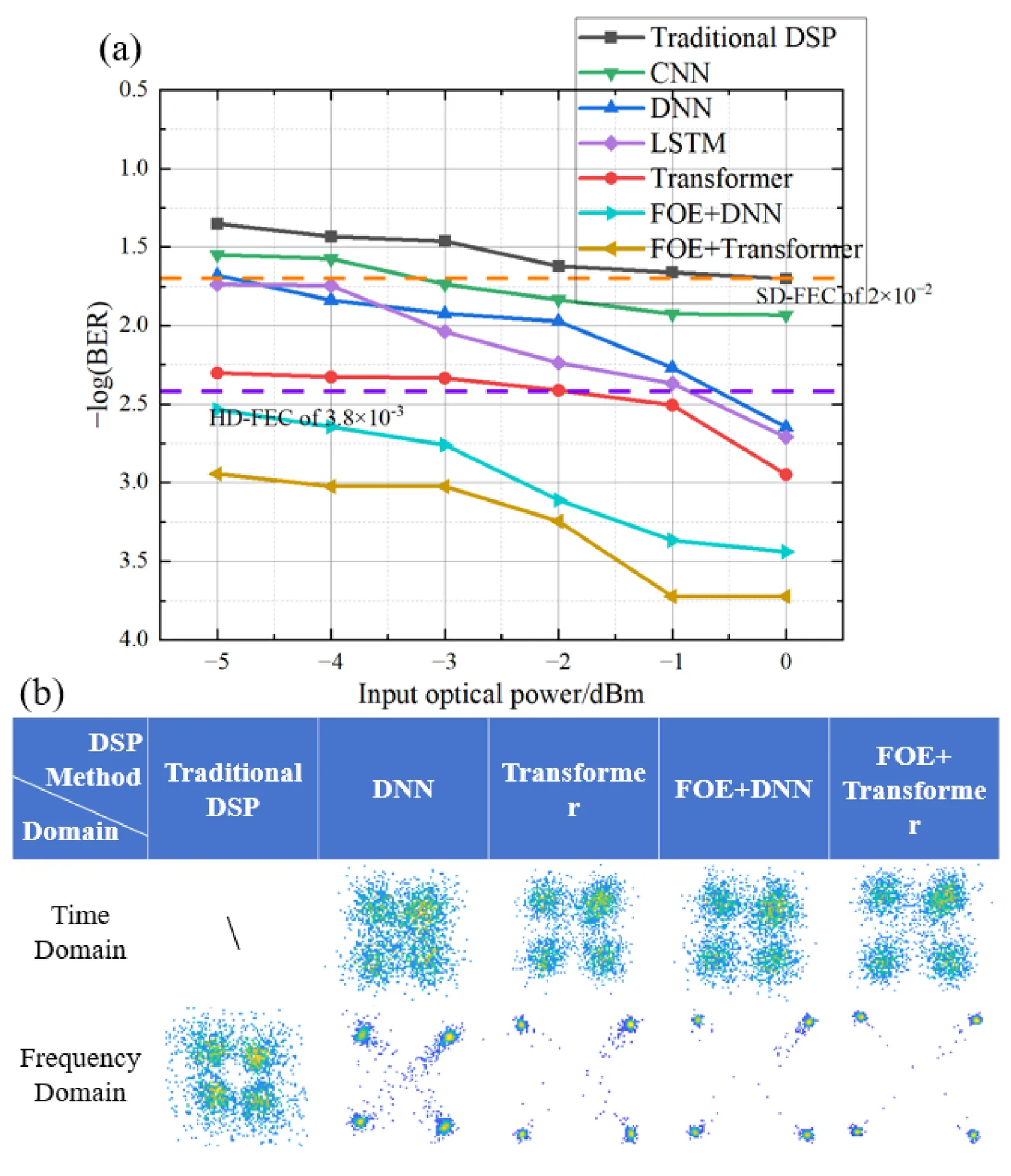
(**a**) BER and (**b**) constellation comparison of traditional DSP, CNN, DNN, LSTM, Transformer-only, FOE-assisted DNN, and the proposed FOE-assisted Transformer.

Figure 6 compares the BER and constellation performance of the receiver DSP methods. Across the measured input optical power range from −5 dBm to 0 dBm, the proposed FOE-assisted Transformer provides the lowest BER.

Approximate values read from the plotted curves illustrate the gain. At 0 dBm input optical power, the BER of traditional DSP is $2\times 10^{-2}$, CNN $1.17\times 10^{-2}$, DNN $2.27\times 10^{-3}$ and LSTM $1.95\times 10^{-3}$, Transformer-only around $1.13\times 10^{-3}$, FOE-assisted DNN around $3.63\times 10^{-4}$, and FOE-assisted Transformer around $1.89\times 10^{-4}$. Furthermore, evaluating the EVM confirms these trends; at 0 dBm input optical power, the EVM of the proposed method is 0.09, compared to 0.15 for the standalone Transformer and 0.14 for the FOE-assisted DNN. Therefore, relative to Transformer-only, the proposed FOE-assisted Transformer reduces the BER by 5.98 times at this operating point. Relative to FOE-assisted DNN, it still provides an additional improvement of 1.92 times.

The constellation diagrams visually confirm the BER curves. Traditional DSP leaves broad and partially overlapping clusters. DNN and Transformer reduce the spreading; FOE-assisted DNN further improves phase alignment; and the proposed FOE-assisted Transformer produces the most compact clusters in both time-domain and frequency-domain comparisons. The result supports the interpretation that FOE removes a global rotation and self-attention suppresses residual local distortion. Additionally, the constellation after the time-domain Transformer mainly reflects waveform-level correction before FFT, so the clusters are partially tightened but may still contain residual subcarrier-domain rotation and ICI. In contrast, the constellation after the frequency-domain Transformer is more compact and better aligned with the ideal decision points because the network directly operates on FFT-output subcarrier symbols and can learn residual amplitude, phase, and neighboring-subcarrier interference after FOE compensation.

### 4.2. Ablation Study

**Figure 7:**
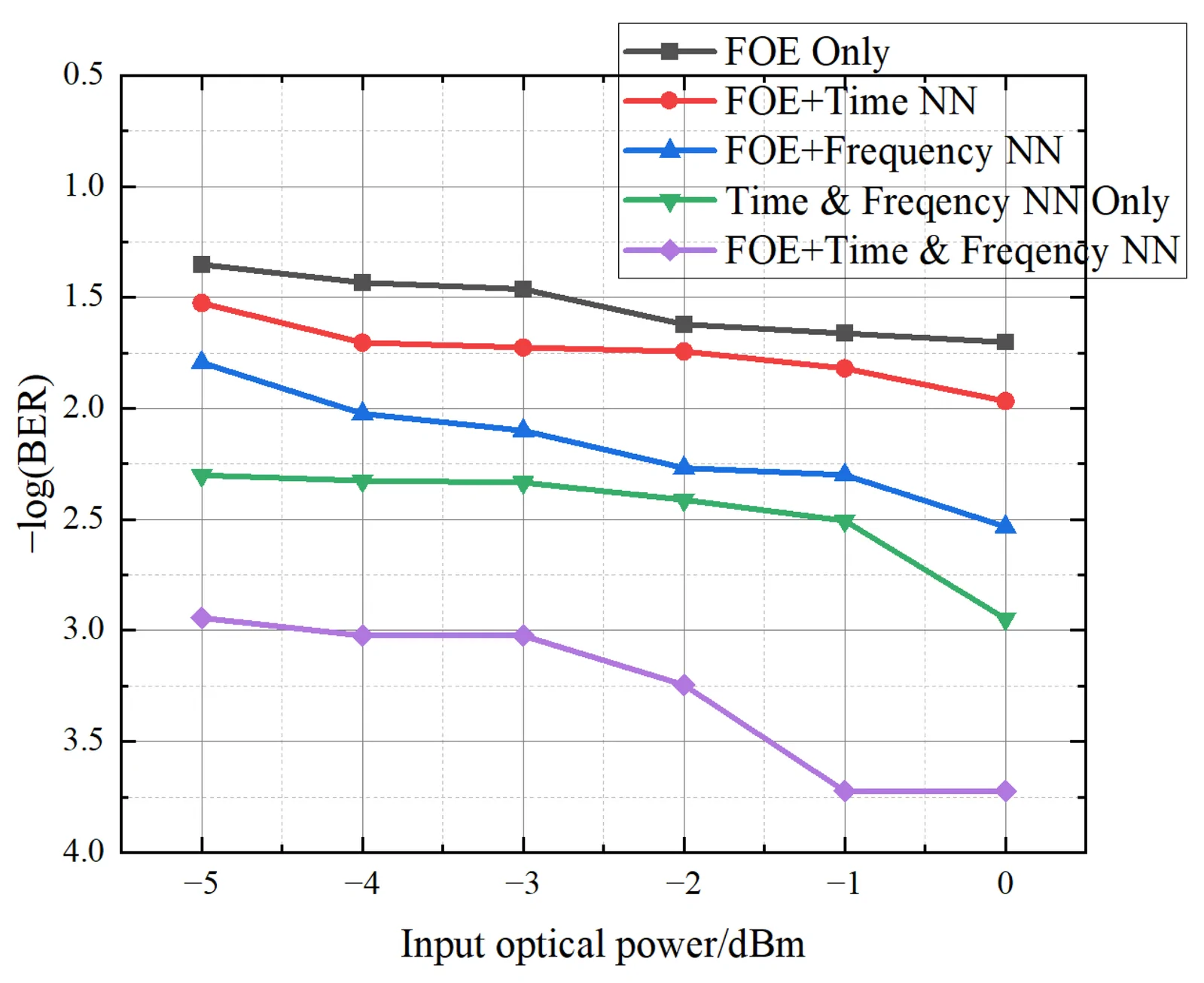
Ablation study comparing FOE, FOE + time-domain Transformer, FOE + frequency-domain Transformer, dual-domain Transformer without FOE, and the complete proposed architecture.

To explicitly demonstrate the necessity of all three processing stages (FOE, time-domain Transformer, and frequency-domain Transformer), an ablation study was conducted, with results summarized in Figure 7.

The FOE alone achieves a BER of 1.99 × 10^−2^. Adding the time-domain Transformer improves the BER to 1.08 × 10^−2^, while using only the frequency-domain Transformer with FOE achieves 2.94 × 10^−3^. The dual-domain Transformer without FOE yields a BER of 1.13 × 10^−3^. Only the complete proposed architecture achieves the optimal BER of 1.89 × 10^−4^ at 0 dBm, confirming that the performance gain stems from the synergistic cooperation between physical tracking and dual-domain attention.

### 4.3. Quantitative CFO Characterization

To further elucidate the role of the FOE module, we quantitatively characterized the CFO under specific experimental conditions. The results indicate that the inherent absolute CFO during transmission fluctuated around 14.25 MHz, corresponding to a steady-state normalized CFO of approximately 8.91 × 10^−4^ relative to the subcarrier spacing. Upon applying FOE, the residual normalized CFO was significantly reduced to approximately 4.13 × 10^−4^. This empirical analysis confirms that while FOE mitigates a portion of the global phase rotation, some residual error remains; the subsequent dual-domain Transformer equalizer is then able to effectively track and compensate for these errors by leveraging its local self-attention windows.

### 4.4. Statistical Reliability

To ensure the statistical robustness of the BER and EVM claims, it is important to note that the performance metrics presented in this study are not derived from a single transient measurement. Instead, the experimental data points presented in Figure 6 represent the average values calculated over 5 independent experimental realizations (captured frames). By inherently averaging the evaluated performance over multiple independent runs, the impact of random noise and instantaneous channel fluctuations is effectively mitigated. This methodological approach confirms the stable convergence, consistency, and reliable steady-state performance of the proposed dual-domain Transformer equalizers under the tested physical conditions.

## 5. Training and Hyperparameter Optimization

The training dataset is constructed offline using known transmitted payloads. During transmission, comb pilots (inserted every eight subcarriers) are primarily utilized for initial frame synchronization and baseline phase tracking, whereas the Transformer equalizers are trained using a supervised MSE criterion on the payload symbols. Table 2 summarizes the specific impairments learned by each Transformer branch, and Table 3 details the training hyperparameters.

### 5.1. Dual-Domain Loss Weighting

The two Transformer branches are trained with a weighted dual-domain mean-square-error loss:

(9) $$L=w_{time}L_{time}+w_{freq}L_{freq}$$

**Figure 8:**
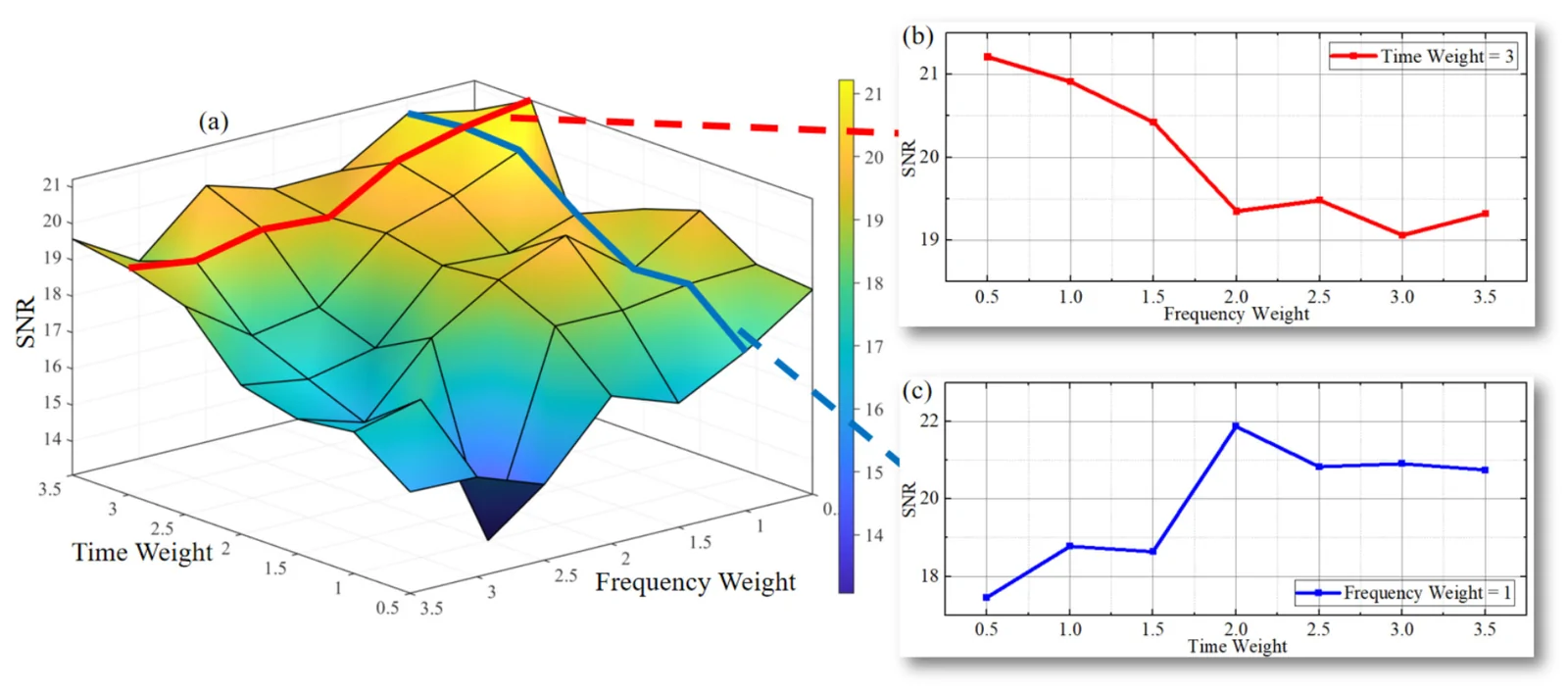
(**a**) Optimization of time-domain and frequency-domain loss weights. The optimum is obtained in a balanced region, with the final setting selected as *w_time_* = 3 and *w_freq_* = 1. More detailed signal SNR variation curves are shown at (**b**) *w_time_* = 3 and (**c**) *w_freq_* = 1.

The sweep in Figure 8 shows that the final SNR depends on the balance between waveform-level and subcarrier-level recovery. If the time-domain weight is too small, the pre-FFT waveform remains insufficiently stabilized; if the frequency-domain weight dominates, the model may overfit subcarrier symbols while sacrificing waveform consistency. The selected setting *w_time_* = 3 and *w_freq_* = 1 emphasizes time-domain preconditioning while preserving frequency-domain refinement.

### 5.2. Convergence

**Figure 9:**
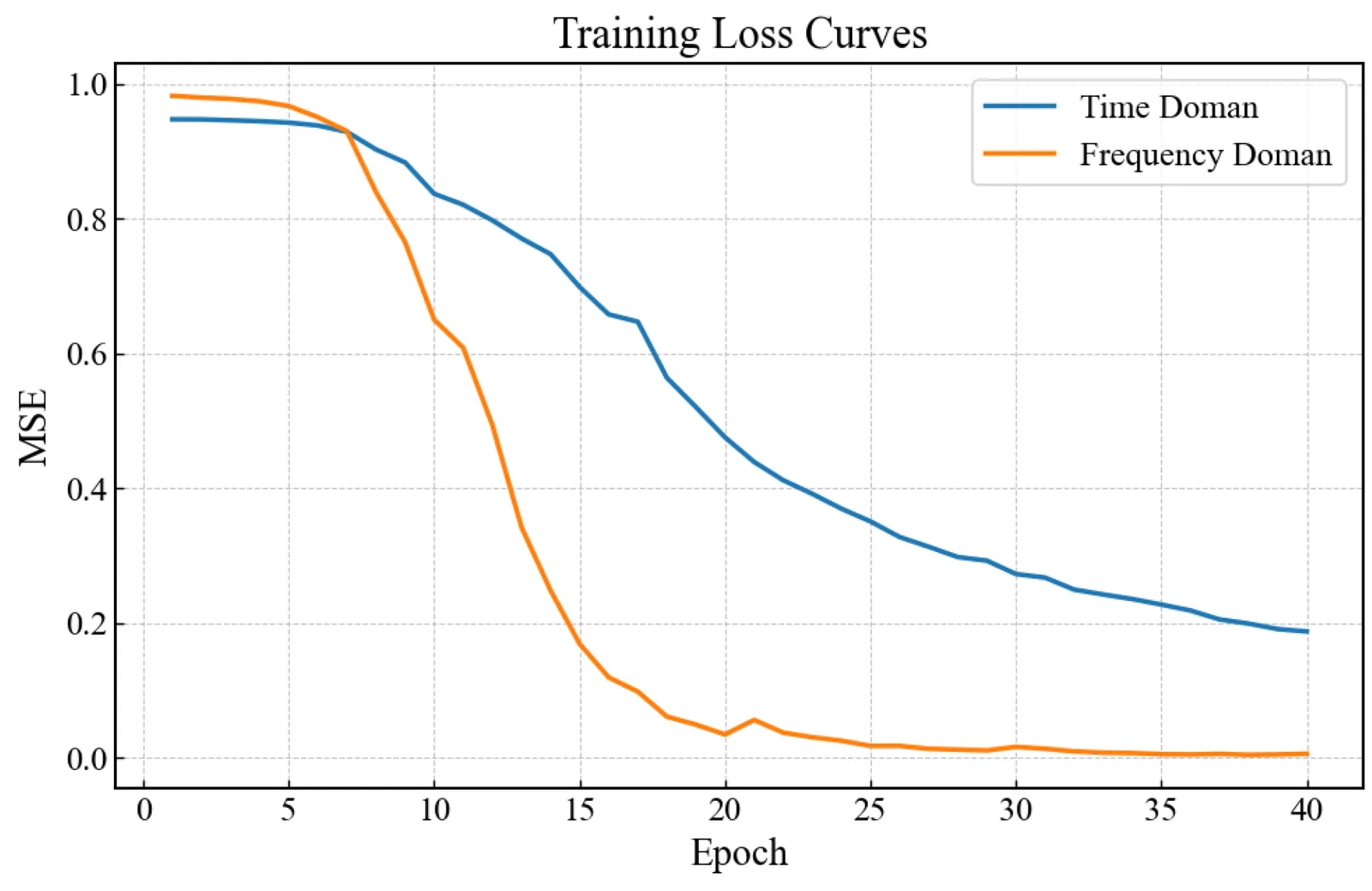
Training loss curves in the time and frequency domains.

The training-loss curves in Figure 9 show that the frequency-domain Transformer converges more quickly than the time-domain Transformer. This is physically reasonable because FFT converts much of the linear channel response into a subcarrier-domain representation, whereas the time-domain branch must handle CP structure, residual timing error, waveform memory, and front-end filtering directly. The different convergence speeds further justify the dual-domain design and the weighted training objective.

## 6. Conclusions

In this work, we experimentally demonstrated an FOE-assisted dual-domain Transformer equalizer in an optical-heterodyne RoF–wireless OFDM transmission system. In the RoF portion, the 16-GBaud OFDM waveform modulates the 1550 nm carrier from ECL-1; this modulated carrier is combined with the 1551.1 nm unmodulated tone from ECL-2 and transported through SMF to a remote UTC-PD. Photonic heterodyne beating in the UTC-PD simultaneously performs optical-to-electrical conversion and generates an approximately 137-GHz D-band OFDM carrier near the transmitting antenna. After electrical amplification, the signal is radiated over a 4.6 km free-space wireless path, down-converted by an electrical I/Q receiver, and captured by a 160-GSa/s real-time oscilloscope. Accordingly, the experiment combines an SMF RoF feeder with a 4.6 km wireless span rather than a 4.6 km fiber span.

For this cascaded RoF–wireless experimental system, we proposed a receiver algorithm that combines CP-correlation-based frequency-offset estimation with compact local-window Transformer equalization in both time and frequency domains. The FOE module first compensates for the dominant CFO-induced global phase rotation arising from the optical-heterodyne and electrical down-conversion stages. The time-domain Transformer then improves the pre-FFT waveform by learning local residual distortion accumulated across the RoF and wireless segments, and the frequency-domain Transformer further refines FFT-output subcarrier symbols by suppressing residual ICI and subcarrier-dependent amplitude/phase errors. This physically ordered combination of model-driven and data-driven processing is the central innovation of the work.

The experimental results confirm the effectiveness of this design. Under comparable MAC operation counts, the proposed FOE-assisted dual-domain Transformer outperforms traditional DSP, DNN, CNN, LSTM, Transformer-only equalization, and FOE-assisted DNN equalization in BER, EVM, and constellation quality. At 0 dBm optical input power, the proposed method achieves a BER of 1.89 × 10^−4^, reducing the BER by about 5.98 times compared with Transformer-only equalization and by about 1.92 times compared with FOE-assisted DNN equalization. The EVM is also reduced to approximately 0.09, and the recovered constellation points become the most compact and best aligned among the compared schemes.

In this paper, by using FOE to remove the dominant global offset and Transformer self-attention to learn the remaining local time-frequency impairments across the RoF feeder, photonic upconverter, wireless channel, and electrical receiver, the method provides a practical path toward robust signal recovery in future millimeter-wave/terahertz RoF fronthaul, fiber-wireless access, and beyond-5G/6G communication systems. While the current experimental demonstration utilizes a 16-GBaud QPSK signal to validate the framework under severe long-reach RoF and wireless channel conditions, the proposed FOE-assisted Transformer architecture is inherently independent of the specific constellation format. By decoupling the deterministic CFO from data-dependent distortions, we anticipate that this equalizer can be generalized to support higher-order modulation formats (such as 16-QAM or 64-QAM) in future deployments, provided the network capacity and training datasets are appropriately scaled.

## Figures and Tables

**Table 1:** Complexity-matched neural equalizer configurations.

Equalizer	Domain	Window	Key Hyperparameters	MACs/Window
Transformer	Time	9	*d* = 32, *h* = 2, *L* = 4, *f* = 16	206,272
	Frequency	11	*d* = 32, *h* = 2, *L* = 4, *f* = 32	302,784
DNN	Time	9	[512, 272, 128, 128]	209,152
	Frequency	11	[512, 400, 128, 96, 96]	300,224
CNN	Time	9	*ch* = 4-64-116, *k* = 3, *p* = 1	207,592
	Frequency	11	*ch* = 4-64-140, *k* = 3, *p* = 1	304,408
LSTM	Time	9	*H* = 52, *layers* = 2	~209,768
	Frequency	11	*H* = 81, *layers* = 1	~303,102

**Table 2:** Impairments addressed by dual-domain transformers.

Domain	Learned Impairments
Time	Waveform-level residual distortion, timing-induced memory, front-end bandwidth effects, CP structure dependencies
Frequency	Residual ICI, subcarrier-dependent phase error, amplitude distortion, local non-linear time-frequency distortions

**Table 3:** Training Hyperparameters.

Hyperparameter	Value
Optimizer	Adam
Learning Rate	2.5 × 10^−3^
Loss Function	MSE
Batch Size	32
Epochs	40

## Data Availability

Data is unavailable due to privacy or ethical restrictions.
